# A Hierarchical Framework for Quadruped Robots Gait Planning Based on DDPG

**DOI:** 10.3390/biomimetics8050382

**Published:** 2023-08-22

**Authors:** Yanbiao Li, Zhao Chen, Chentao Wu, Haoyu Mao, Peng Sun

**Affiliations:** 1 College of Mechanical Engineering, Zhejiang University of Technology, Hangzhou 310023, China; lybrory@zjut.edu.cn (Y.L.); 2112002350@zjut.edu.cn (Z.C.); 2112102096@zjut.edu.cn (C.W.); 2111902111@zjut.edu.cn (H.M.); 2 Key Laboratory of Special Purpose Equipment and Advanced Processing Technology, Ministry of Education and Zhejiang Province, Zhejiang University of Technology, Hangzhou 310023, China; 3 Huzhou Institute of Digital Economy and Technology, Zhejiang University of Technology, Huzhou 313000, China

**Keywords:** quadruped robots, hierarchical reinforcement learning, Deep Deterministic Policy Gradient, model predictive control

## Abstract

In recent years, significant progress has been made in employing reinforcement learning for controlling legged robots. However, a major challenge arises with quadruped robots due to their continuous states and vast action space, making optimal control using simple reinforcement learning controllers particularly challenging. This paper introduces a hierarchical reinforcement learning framework based on the Deep Deterministic Policy Gradient (DDPG) algorithm to achieve optimal motion control for quadruped robots. The framework consists of a high-level planner responsible for generating ideal motion parameters, a low-level controller using model predictive control (MPC), and a trajectory generator. The agents within the high-level planner are trained to provide the ideal motion parameters for the low-level controller. The low-level controller uses MPC and PD controllers to generate the foot-end force and calculates the joint motor torque through inverse kinematics. The simulation results show that the motion performance of the trained hierarchical framework is superior to that obtained using only the DDPG method.

## 1. Introduction

In contrast to their wheeled and tracked counterparts, quadruped robots confer two pronounced mobility advantages: discrete footholds and the capacity for multi-degree-of-freedom limb movements. The first advantage stems from the ability of quadruped robots to adeptly navigate intricate terrains, adroitly surmounting obstacles and spanning gaps, all while exerting minimal ground pressure. The second advantage resides in quadruped robots’ quadrilateral configuration, with each limb endowed with diverse degrees of freedom. This architecture empowers them to manifest a diverse spectrum of motion patterns and behaviors. Quadruped robots can dynamically regulate their body height, proactively dampen vibrations, and adroitly manipulate objects using their limbs, thereby significantly enhancing their overall flexibility [1]. These merits have collectively fueled exploration across a multitude of application scenarios for quadruped robots, progressively steering the research attention toward pragmatic, real-world implementations [2,3,4], such as deploying robots in intricate contexts like terrain surveys and disaster response [5,6,7]. This transformative shift underscores the escalated requisites placed upon the realm of intelligent robot control.

In recent years, there has been a burgeoning interest in quadruped robot research, encompassing diverse domains like industrial applications and robot education. The control strategies applied to these robots can be broadly categorized into two classes: conventional control methods and reinforcement-learning (RL)—based approaches. Notably, conventional control methods, including model predictive control (MPC) [8,9], have found extensive application. In contrast, reinforcement learning (RL)—based techniques empower agents to acquire and enhance their performance from scratch, devising policies to attain specific objectives. This paradigm facilitates heightened adaptability and has gained notable prominence in the arena of legged robot control. Recent investigations consistently underscore the ascendancy of data-driven RL methodologies in adeptly governing legged robots’ behavior [10,11,12,13].

However, a significant challenge arises in the context of quadruped robots due to their continuous states and extensive action space. The continuous states of quadruped robots necessitate the continual processing and interpretation of uninterrupted sensory inputs. These inputs encompass crucial parameters such as position, velocity, and orientation. This processing is essential to accurately determine the robot’s current state, which forms the foundation for subsequent decision-making. Concurrently, the extensive action space available to quadruped robots equips them with a broad spectrum of potential actions. These actions span from diverse gaits to nuanced variations in leg positions and joint torques. However, the sheer abundance of feasible actions gives rise to a phenomenon known as combinatorial explosion. This phenomenon poses a formidable challenge, obstructing the systematic exploration and optimization of the most optimal set of actions [14,15]. The continuous nature of the states and the expansive action space collaboratively introduce intricacies into the decision-making process. Consequently, this complexity necessitates the deployment of efficient algorithms and substantial computational resources to adeptly manage the high-dimensional state and action domains.

To address these formidable challenges, researchers have employed diverse strategies, encompassing techniques like predefined signals and action-reference-guided learning [16]. Another avenue exhibiting considerable promise is hierarchical reinforcement learning (HRL). This paradigm decomposes intricate tasks into more manageable sub-tasks, facilitating the accelerated acquisition of targeted strategies. Notably, HRL has exhibited efficacy in governing quadruped robots, culminating in the development of self-learning hierarchical frameworks that showcase remarkable control proficiency [17]. In a related work [14], the hierarchical architecture and reinforcement learning framework inherent in HRL demonstrated favorable learning efficiency and adeptness in adapting to varying tasks. However, it is important to acknowledge that this method might necessitate a substantial corpus of training data and could present challenges associated with the parameter configuration and fine-tuning. An alternative pioneering solution directed at surmounting the gait generation challenge within the realm of quadruped robots is detailed in. This inventive approach amalgamates HRL with the Soft-Actor-Critic (SAC) algorithm, enabling the robot to not only acquire and optimize intricate gaits but also retain the adaptability required to accommodate diverse environmental and task scenarios. Nonetheless, it is imperative to underscore that issues related to the complexity of training and the capacity for generalization remain subjects warranting further attention.

This paper introduces a stratified framework in which the upper-level planning layer furnishes motion parameters to the lower-level control layer. Following this, the lower-level control layer computes the relevant motor torque predicated on the input parameters, thereby attaining meticulous robot control. This methodology efficiently diminishes the exploration range at the upper level, thereby expediting real-time adaptations and revisions to accommodate alterations and uncertainties in the system. Consequently, it amplifies the training efficiency and bolsters the resilience of the system.

To establish the forward kinematics of the quadruped robot, this paper employs the D–H modeling method, enabling the derivation of the single-leg Jacobian matrix and the analysis of the simplified dynamic model. The proposed HRL framework consists of a high-level planner that utilizes the Deep Deterministic Policy Gradient (DDPG) method to generate the optimal motion parameters for the robot. At the lower level, the controller employs the MPC method and composite trajectory planning method to address the foot-end forces during the support and swing phases and calculate the output torque for each joint motor. The motion performance of both the DDPG method and the proposed hierarchical method during training is analyzed using Matlab/Simulink software. This analysis serves to validate the effectiveness of the hierarchical deep reinforcement learning (DRL) model and acts as a reference for subsequent motor selection and optimization of actual prototypes.

## 2. Kinematic and Dynamic Analyses

Kinematics elucidates the geometric correlation between the robot’s end effector and the position of each joint, whereas dynamics elucidates the mechanical interplay between joint positions and torques. These two disciplines serve as the cornerstone for comprehensively studying robots.

### 2.1. Kinematic Analysis

The experimental platform chosen for this study is the Spot-Micro, a four-legged robot model consisting of a body and four legs. Each leg possesses a 3-degree-of-freedom hinge structure comprising a rolling hip joint, a pitching hip joint, and a pitching knee joint.

**Figure 1:**
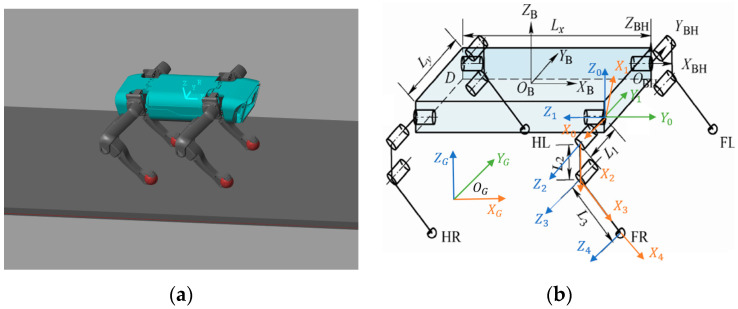
Three-dimensional models of the quadruped robot: (**a**) physical model of the robot; (**b**) simplified model of the robot.

To depict the functional relationship between the position and orientation of the leg end and the center of the robot’s body, the D–H modeling method is employed to establish the corresponding D–H model. Physical and simplified representations of the model are illustrated in Figure 1a,b.

As shown in Figure 1b, the {B} coordinate system is the centroid coordinate system of the quadruped robot. The coordinate system {B} is projected onto the support plane to obtain the world reference coordinate system {G}. The {0} coordinate system is fixed on the rolling hip joint, with the Z_0_ axis pointing vertically upwards. The {1} coordinate system is fixed on the rolling hip joint and rotates with it, with the Z_1_ axis pointing towards the left. The {2} coordinate system is fixed on the pitching hip joint and rotates accordingly, with the Z_2_ axis pointing out of the plane of the paper. The {3} coordinate system is fixed on the pitching knee joint and rotates with it, with the Z_3_ axis pointing out of the plane of the paper. The {4} coordinate system is fixed on the foot end and moves with the lower leg, with the Z_4_ axis pointing out of the plane of the paper. 

According to the relationship between the above coordinate systems, the D–H parameter table of a single leg of the quadruped robot is shown in Table 1.

In the above table, $a_{i}$ is the distance from ${\hat{Z}}_{i}$ to ${\hat{Z}}_{i+1}$ along ${\hat{X}}_{i}$, $\alpha_{i}$ is the angle of rotation from ${\hat{Z}}_{i}$ to ${\hat{Z}}_{i+1}$ along ${\hat{Z}}_{i+1}$, $d_{i}$ is the distance from ${\hat{X}}_{i-1}$ to ${\hat{X}}_{i}$ along ${\hat{Z}}_{i}$, and $\theta_{i}$ is the angle of rotation from ${\hat{X}}_{i-1}$ to ${\hat{X}}_{i}$ along ${\hat{Z}}_{i}$.

Taking the left front leg as an example, the transformation matrix from the centroid to the foot-end coordinate system of the quadruped robot is derived as follows:

(1) TB4=[RP01]

where R is the rotation matrix of the foot coordinate system relative to the body coordinate system.

(2) $$R=\left[\begin{array}{ccc} s_{23} & c_{23} & 0\\ -s_{1}c_{23} & s_{1}s_{23} & -c_{1}\\ -c_{1}s_{23} & c_{1}s_{23} & s_{1} \end{array}\right]$$

where P is the position relationship between the foot end and the robot centroid.

(3) $$P=\left[\begin{array}{c} P_{x}\\ P_{y}\\ P_{z} \end{array}\right]=\left[\begin{array}{c} L_{3}s_{23}+L_{2}s_{2}+\frac{L_{x}}{2}\\ -s_{1}(L_{3}c_{23}+L_{2}c_{2})-L_{1}c_{1}+\frac{L_{y}}{2}\\ -c_{1}(L_{3}c_{23}+L_{2}c_{2})+L_{1}s_{1} \end{array}\right]$$

According to the above coordinate system relationship, the Jacobian matrix of a single leg can be obtained by computing the partial derivatives of the forward kinematics equations with respect to the joint angles for each joint.

(4) $$J(\theta )=\left[\begin{array}{ccc} 0 & L_{2}c_{2}+L_{3}c_{23} & L_{3}c_{23}\\ -c_{1}(L_{3}c_{23}+L_{2}c_{2})+L_{1}s_{1} & s_{1}(L_{3}s_{23}+L_{2}s_{2}) & L_{3}s_{1}s_{23}\\ s_{1}(L_{3}c_{23}+L_{2}c_{2})+L_{1}c_{1} & c_{1}(L_{3}s_{23}+L_{2}s_{2}) & L_{3}c_{1}s_{23} \end{array}\right]$$

where $s_{ab}$ and $c_{ab}$ represent $\sin(\theta_{a}+\theta_{b})$ and $\cos(\theta_{a}+\theta_{b})$, respectively. The specific parameters of the quadruped robot are shown in Table 2.

### 2.2. Dynamic Analysis

Due to the inherent complexity of dynamic models of quadruped robots, some simplifications are necessary [18]. In this study, the robot model is simplified to a single-rigid-body model, where the majority of the mass is concentrated in the torso, and the legs contribute only to a small extent. The model neglects the influence of leg swinging on the center of mass and focuses solely on the impact of the supporting leg force on the center of mass. The dynamic model can be expressed as follows:

(5) $$\left[\begin{array}{ccc} I_{3} & \cdots & I_{3}\\ (r_{1}-p_{com})\times & \cdots & (r_{4}-p_{com})\times \end{array}\right]u_{k}=\left[\begin{array}{c} m({\ddot{p}}_{com}+g)\\ I_{g}{\dot{\omega }}_{b} \end{array}\right]$$

where *m* and $I_{g}$ are the robot mass and inertia, g is gravity, *r_i_* is the corresponding position of the foot-end legs, $p_{com}$ is the position of the center of mass, $u_{k}=[F_{1},F_{2},F_{3},F_{4}]\in {\mathbb{R}}^{12}$ is the vector of the Cartesian forces for the four feet, and ${\dot{\omega }}_{b}$ is the angular velocity of the trunk rotation.

## 3. Hierarchical Framework

This section provides a comprehensive description of the hierarchical framework, which consists of two components: the high-level planner and the low-level controller. The high-level planner is designed based on the DDPG algorithm, while the low-level controller consists of MPC and a trajectory generator.

### 3.1. Algorithm Overview

**Figure 2:**
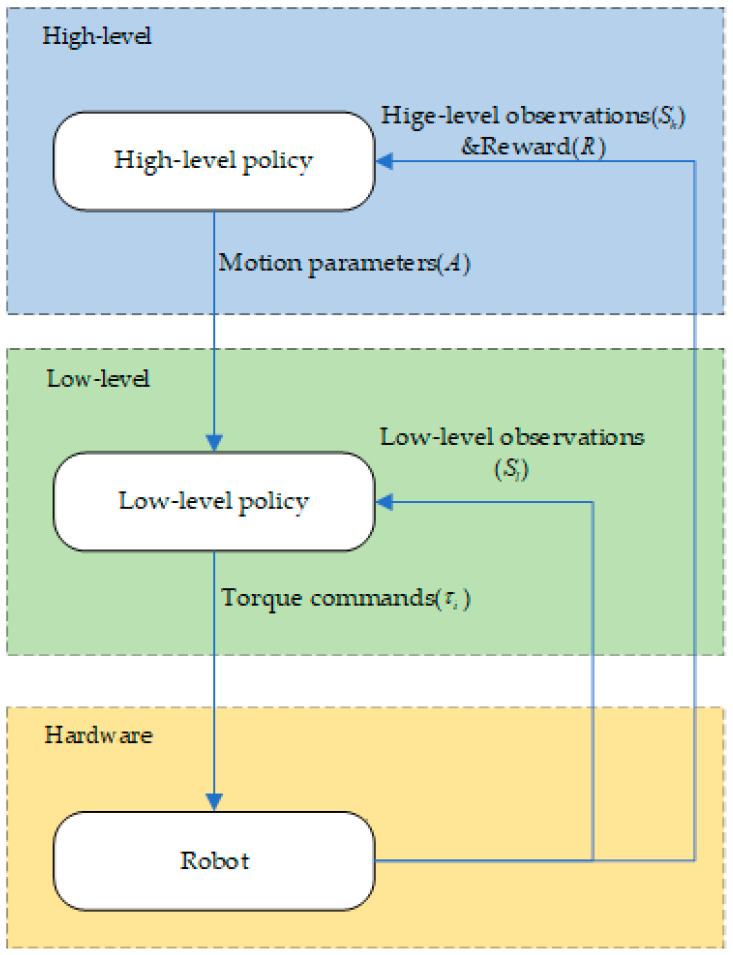
The proposed hierarchical reinforcement learning framework. The high-level planner receives high-level observations $S_{h}$ and outputs the ideal motion parameters A. The low-level control layer accepts high-level motion parameters A and low-level observation values $S_{i}$ and calculates the torque of each motor $\tau_{i}$.

The HRL framework is shown in Figure 2. 

The high-level planner operates by receiving high-level observations ($S_{h}$) at a specified frequency. These observations consist of the position, orientation, and velocity of the robot’s centroid. Based on these inputs, the high-level planner generates motion parameters (A) that are subsequently relayed to the low-level controller.

The low-level controller, in turn, receives both low-level observations ($S_{i}$) and the motion parameters (A) from the high-level planner. The low-level observations encompass data from the inertial measurement unit (IMU), including the roll angle, pitch angle, roll angular velocity, and pitch angular velocity. Utilizing this information in conjunction with the motion parameters, the low-level controller computes the required torques ($\tau_{i}$) for each joint motor, which are then sent to the hardware for execution.

This process continues iteratively within the framework, with subsequent observations and reward values being obtained after each interaction. For a more detailed understanding, please refer to Algorithm 1.
**Algorithm 1.** Executing a Hierarchical Policy**Require:** Initialize replay buffer Initialize network parameters; 1: **for** 0 < epochs < N maxepochs **do** 2:  Initialize observation, action, etc.; 3:  **for** 0 < step < N maxsteps **and** not reach termination conditions **do** 4:   The high-level network receives high-level observations and outputs motion parameters 5:   The low-level controller receives the motion parameters and low-level observations, and outputs the required torque for each motor 6:   Run robot to obtain the next observation; 7:   Get reward; 8:   Store memory; 9:   Update network 10:  **If** step = N maxsteps **or** reach termination conditions **then** 11:    Calculate the total reward value 12:    Send messages to reset environment and robot position; 13:  **End** 14:  **End** 15: **End**

### 3.2. High-Level Planner

In the hierarchical framework, the high-level planner is responsible for receiving the body state variables and planning the motion parameters of the support leg and swing leg. 

RL requires adherence to a Markov decision process (MDP), which represents the environment as a tuple (s,a,p,r,α). It includes the state s, action a, transition probability of the state p, reward r, and discount factor α. The DDPG algorithm is proposed to solve continuous action control problems; this algorithm has demonstrated excellent performance in quadruped robot control tasks [19,20,21]. This paper employs the DDPG algorithm as the high-level planner to determine the optimal action for each state, maximizing the cumulative reward. 

The algorithm employed in this study can be compartmentalized into three core segments: initialization, interaction, and update. During the runtime, the network parameters and the replay buffer are primed in the initial phase. The DDPG algorithm amalgamates elements from the Deep Q-Network (DQN) algorithm and the Actor–Critic framework. This amalgamation involves four distinct networks: a critic network coupled with its corresponding target network, in conjunction with an actor network accompanied by its corresponding target network. Within the scope of this research, the DDPG algorithm encompasses four neural networks: the actor network, critic network, target actor network, and target critic network. Each of these neural networks is composed of two concealed layers, with one comprising 512 neurons and the other comprising 256 neurons. The activation function for each layer is executed using the rectified linear unit (ReLU) function.

In each episode, noise is injected to augment the interaction with the environment. The data acquired from these interactions are preserved in the replay buffer. When the time for network updates arrives, a minibatch of data is chosen at random from the buffer. Subsequently, the critic network, actor network, and their respective target networks undergo consecutive updates. This iterative update procedure persists for every episode until the algorithm reaches its termination point.

A random minibatch of N transitions $\left(s_{t},a_{t},r_{t},s_{t+1}\right)$ from the replay buffer is sampled. First, the target value $y_{t}=r_{t}+\alpha Q^{\prime }(s_{t+1},\omega^{\prime }(s_{t+1}|\theta_{Q^{\prime }}))$ is calculated using the $Q^{\prime }$ value obtained from the target critic network, where α is the discount factor, which indicates the depreciation degree of future rewards and is set to 0.95. Then, a loss function is constructed by utilizing the mean square deviation between the target value $y_{t}$ and the current Q value. Its parameter $\theta_{Q}$ is updated by minimizing the loss:

(6) $$J(\theta_{Q})=\frac{1}{N}\sum_{t=1}^{N}{\left(y_{t}-Q(s_{t},a_{t}|\theta_{Q})\right)}^{2}$$

The actor network is mainly responsible for generating actions *a_t_* based on the current state *s_t_*, interacting with the environment to generate rewards *r_t_*_+1_ and new states *s_t_*_+1_. The parameter $\theta_{\pi }$ of the actor policy is updated using the sampled policy gradient:

(7) $$\nabla_{\theta_{\pi }}J=\frac{1}{N}\sum_{t=1}^{N}\left[\nabla_{a}Q(s_{t},a_{t}|\theta_{Q}){\left|{}_{s=s_{t},a=\pi (s_{t})}\nabla_{\theta \pi }\pi (s|\theta_{\pi })\right|}_{s=s_{t}}\right]$$

In order to ensure more stable training, periodic soft updates are performed on the target networks. The update functions for the target critic network parameter $\theta_{Q^{\prime }}$ and target actor network parameter $\theta_{\pi^{\prime }}$ are as follows:

(8) $$\theta_{Q^{\prime }}=\gamma \theta_{Q}+(1-\gamma )\theta_{Q^{\prime }}$$

(9) $$\theta_{\pi^{\prime }}=\gamma \theta_{\pi }+(1-\gamma )\theta_{\pi^{\prime }}$$

where γ is the set learning rate, set as 0.001.

In this paper, the high-level observations ($S_{h}$) consist of various state variables. These variables encompass the velocity of the center of mass along the X, Y, and Z axes, with the quaternion representation defining the orientation of the torso and the yaw, pitch, and roll angles of the torso. Additionally, the torque output of each joint motor from the previous time step is included. Furthermore, variables that are indirectly related to the reward function but exert an influence on the motion of the quadruped robot are integrated. These comprise the center-of-mass position along the Y and Z axes, the angle and angular velocity of each leg motor, and the normal and frictional forces arising from each leg’s ground contact.

Overall, the selection of high-level observations encompasses a total of 56 state variables, encompassing both directly pertinent and indirectly influential information. To mitigate the learning complexity inherent in DRL and to enhance the training efficiency, these state variables undergo normalization prior to their incorporation into the neural network. By constraining all state variables within the range of (−1, 1), effective learning facilitation is achieved.

The reward function was designed to encourage a faster velocity and longer duration while penalizing deviations from the desired direction and excessive torque. The reward function is represented as follows:

(10) $$r_{t}=3v_{x}+30\frac{T_{s}}{T_{f}}-10\theta^{2}-0.01\sum_{i}\tau_{t-1}^{i}{}^{2}$$

where $v_{x}$ is the velocity along the X axis in the world coordinate system, $T_{f}$ is the duration, $T_{s}$ is the set time, $\tau_{t-1}^{i}$ denotes the torque output of the motor in the previous time step, and θ represents the yaw angle of the robot.

Previous studies have demonstrated that setting termination conditions can improve the efficiency of learning. In this study, the cessation criteria are delineated as follows. The experiment will be halted if any of the subsequent conditions are met: the yaw angle, roll angle, or pitch angle exceeds a predetermined threshold; the center-of-mass position descends below a specified value; or any joint of the robot comes into contact with the ground.

The high-level planning layer in the framework is responsible for providing several motion parameters to the lower-level control layer. These include the forward velocity ($v_{x}$) of the center of mass for the supporting legs and the walking leg length (S) and lifting leg height (H) for the swinging legs. Furthermore, the high-level layer also outputs the execution time Δt for the low-level layer. After the execution cycle concludes, the high-level layer receives new state variables and rewards and subsequently outputs a fresh set of motion parameters for the next iteration.

### 3.3. Low-Level Controller

The low-level controller consists of an MPC controller for the support legs and a trajectory generator for the swing legs. Considering the stability of motion, this paper uses a trot as the robot gait, and the diagonal legs of the quadruped robot are set as groups; that is, the left front leg and the right rear leg are a group, and the left rear leg and the right front leg are a group. The legs of the same group are in the same state at the same time, and the torque input from the corresponding motor is the same. The state of each leg is marked as swinging or supporting, which determines which method to use to calculate its foot force.

#### 3.3.1. Support Leg Controller

The MPC functions on the premise of real-time calculation of an open-loop optimization problem. It leverages the contemporaneous measured data at each sampling interval to construct the optimization problem spanning a predefined time horizon. Subsequently, the initial component of the optimized solution is executed on the controlled entity. This iterative process is reiterated during ensuing sampling instances, with the optimization problem being refreshed employing the novel measured data as the initial states. The fundamental aim of MPC is to prognosticate the forthcoming dynamics of the control system and adeptly steer the controller’s behavior.

The MPC algorithm consists of three main steps. Firstly, it involves predicting the future dynamic changes in the system based on the current measured data. Secondly, an open-loop optimization problem is solved using numerical methods. Finally, the first element or part of the optimized solution is applied to the system. These steps are iteratively performed at each sampling time. Regardless of the model used, the current measured data are used as the initial condition for predicting the future dynamics of the system.

The optimization objective in MPC is to minimize the optimal trajectory error and foot internal force. Based on Equation (5), the optimization objective is constructed using MPC to achieve the desired control performance.

(11) $$\begin{array}{l} \underset{x,u}{\min}\sum_{k=0}^{n-1}\left\|x_{k+1}-x_{k+1,ref}\right\|Q+\left\|u_{k}\right\|R\\ \begin{array}{cc} s.t. & \begin{array}{l} \begin{array}{cc} x_{k+1}=A_{k}x_{k}+B_{k}u_{k}+G & k=1,2,\ldots ,n-1 \end{array}\\ \begin{array}{cc} \underline{c_{k}}\leq C_{k}u_{k}\leq \bar{c_{k}} & k=1,2,\ldots ,n-1 \end{array} \end{array} \end{array} \end{array}$$

where Q and R are diagonal matrices in the cost function, k is the number of state iterations, $x_{ref}$ is the expected state of the centroid obtained from the high-level input, the inverse of $u_{k}\in {\mathbb{R}}^{12}$ is the force exerted by the support leg on the ground, and $\underline{c_{k}}\leq c_{k}u_{k}\leq \bar{c_{k}}$ to keep the support and friction force within the maximum and minimum amplitudes.

(12) $$A_{k}={\left[\begin{array}{cccc} 1_{3} & 0_{3} & R_{z}(\gamma )\Delta t & 0_{3}\\ 0_{3} & 1_{3} & 0_{3} & 1_{3}\Delta t\\ 0_{3} & 0_{3} & 1_{3} & 0_{3}\\ 0_{3} & 0_{3} & 0_{3} & 1_{3} \end{array}\right]}^{k}$$

(13) $$B_{k}={\left[\begin{array}{ccc} 0_{3} & \cdots & 0_{3}\\ 0_{3} & \cdots & 0_{3}\\ I_{g}{}^{-1}[r_{1}-p_{com}]\Delta t & \cdots & I_{g}{}^{-1}[r_{4}-p_{com}]\Delta t\\ 1_{3}\Delta t/m & \cdots & 1_{3}\Delta t/m \end{array}\right]}^{k}$$

(14) $$G=\left[\begin{array}{c} 0_{3}\\ 0_{3}\\ 0_{3}\\ g \end{array}\right]$$

where $R_{z}(\gamma )$ is the rotation matrix of the yaw angle.

#### 3.3.2. Swing Leg Controller

To minimize the impact on the joints during the lifting and landing phases and ensure a smooth foot trajectory, a defined foot trajectory is employed in this paper.

(15) $$\begin{array}{l} \{\begin{array}{c} x=S[\frac{t}{\Delta t}-\frac{1}{2\pi }\sin(\frac{2\pi t}{\Delta t})]\\ z=H[es(\frac{\Delta t}{2}-t)(\frac{t}{\Delta t}-\frac{1}{2\pi }\sin(\frac{4\pi t}{\Delta t})-1)+1] \end{array}\\ es(\frac{\Delta t}{2}-t)=\{\begin{array}{c} \begin{array}{cc} 1 & 0\leq t<\frac{\Delta t}{2} \end{array}\\ \begin{array}{cc} -1 & \frac{\Delta t}{2}\leq t<\Delta t \end{array} \end{array} \end{array}$$

**Figure 3:**
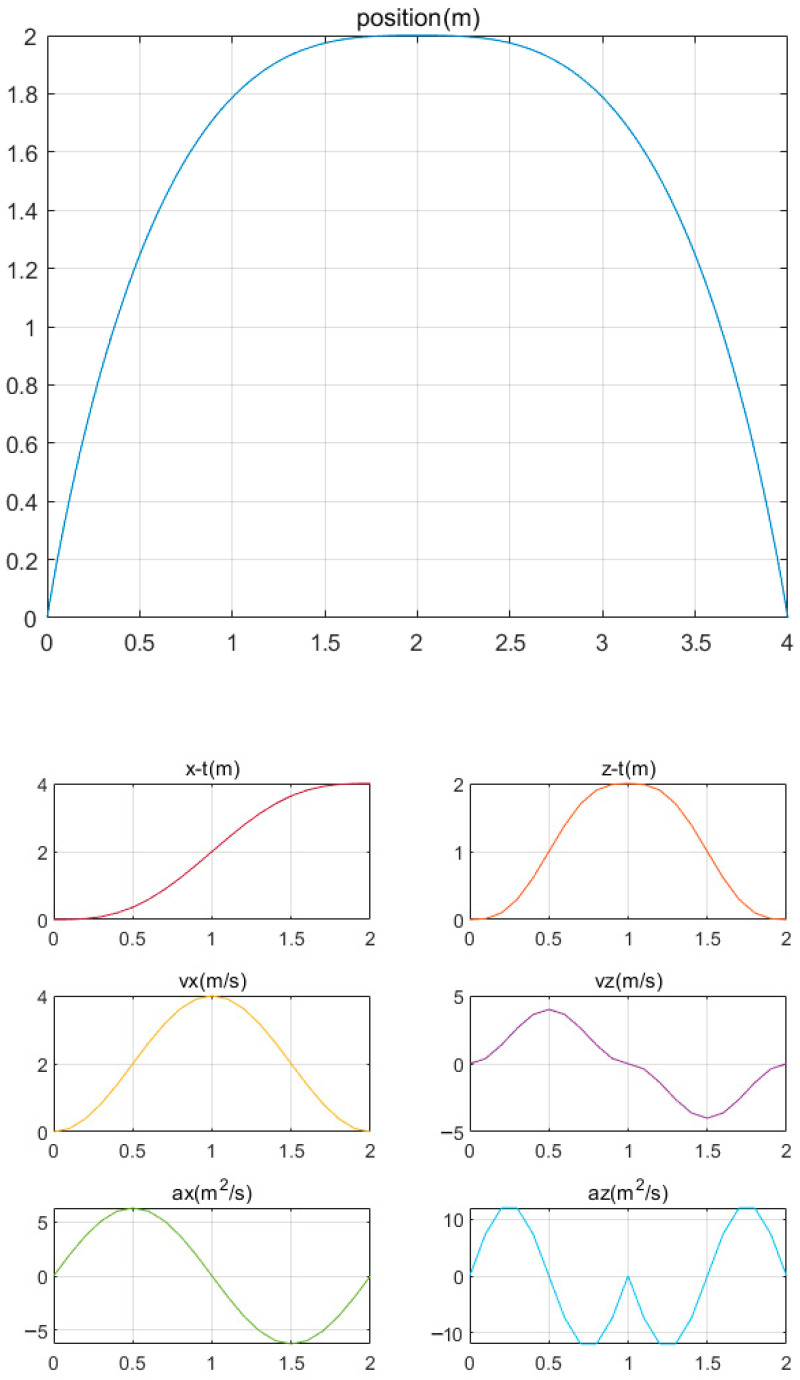
Foot trajectory and displacement, velocity, and acceleration curves.

Upon receiving the swing leg motion parameters, namely the step length (S) and swing foot height (H), from the high-level input, the trajectory generator calculates the foot trajectory. The displacement, velocity, and acceleration during the swing period are depicted in Figure 3.

Figure 3 illustrates that the velocity and acceleration of the swinging leg exhibit continuity throughout one cycle. The velocity–time curve in the XZ-axis direction displays a smooth variation without sudden changes over time. Similarly, the acceleration in the XZ-axis direction remains relatively smooth, with no abrupt change points, and is maintained within a relatively low range. Notably, the velocity and acceleration both reach zero at the start and end time points.

Once the foot trajectory is obtained, the PD controller calculates the real-time swing foot forces.

(16) $$F_{i}=K_{p}(p_{d,i}-p_{i})+K_{d}({\dot{p}}_{d,i}-{\dot{p}}_{i})$$

where $K_{p}$ is the scale factor, $K_{d}$ is the differential coefficient, $p_{d,i}$ is the desired foot-end position of leg *i*, and $p_{i}$ is the current foot-end position of leg i.

By solving the MPC problem and tracking the foot tip trajectory, the foot contact forces of the supporting leg and the swinging leg are obtained. The torque of each joint in a single leg can be calculated by utilizing the principle of virtual work and employing the Jacobian matrix from Equation (3). These torque values are subsequently inputted into the hardware to facilitate robot control.

(17) $$\tau_{d}^{leg_{i}}={\left(J^{i}\right)}^{T}F_{i}$$

where $\tau_{d}^{leg_{i}}$ is a vector composed of the torque output from the motor of joint i, $J_{C}^{i}$ is the Jacobian matrix of leg i, and $F_{i}$ is the desired foot-end force, which is determined by the supporting leg part in $-u_{k}$ calculated using Formula (11) and the swinging leg $F_{i}$ calculated using Formula (16). This is updated at 500 Hz.

## 4. Results

The robot underwent training using Simulink to simulate its walking behavior on flat terrain, evaluating the HRL architecture. Two sets of comparative experiments were conducted. In the first set, solely the DDPG algorithm was employed for training, with the DDPG directly generating the joint motor torques for robot control (referred to as Group_0). In the second set, training was conducted using the hierarchical framework (referred to as Group_1). A total of 4000 training cycles were executed, with each cycle limited to a maximum duration of 10 s. The specific parameters of the robot can be found in Table 3.

**Figure 4:**
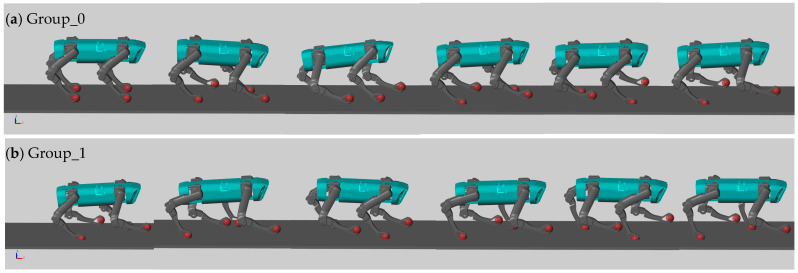
Simulation performance of quadruped after training in Simulink. (**a**) Group_0. (**b**) Group_1.

Figure 4 depicts the gait cycles observed in the two experimental groups. The results clearly demonstrate that Group_0, trained solely with the DDPG algorithm, achieved locomotion. However, due to the extensive search space and the agent’s emphasis on maintaining body balance, the motion performance was suboptimal. Conversely, Group_1, trained using the hierarchical framework proposed in this study, effectively learned to execute a trot gait.

**Figure 5:**
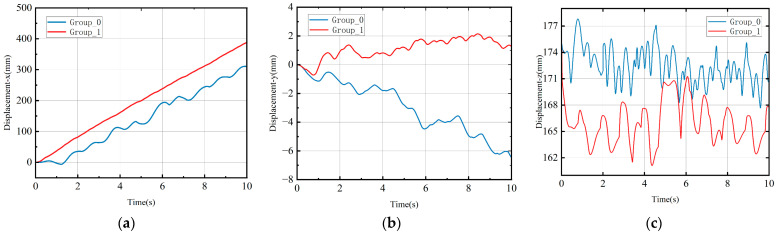
Centroid displacement curve. (**a**) X-axis displacement. (**b**) Y-axis displacement. (**c**) Z-axis displacement.

For a comprehensive analysis of motion in the two experimental groups, the variations in the robot’s center of mass in the XYZ-axis directions were recorded throughout the entire gait cycle, as shown in Figure 5. In the X-axis direction, Group_0 experienced a deceleration in speed and even moved backward due to the impact of the opposite direction. Conversely, Group_1 initially accelerated and subsequently maintained a nearly constant forward velocity. In the Y-axis direction, Group_1 controlled the displacement offset within a relatively small range, while the offset of Group_0 tended to diverge. In the Z-axis direction, it is evident that Group_0 exhibited more frequent fluctuations, while Group_1 demonstrated a periodic up-and-down oscillation pattern.

**Figure 6:**
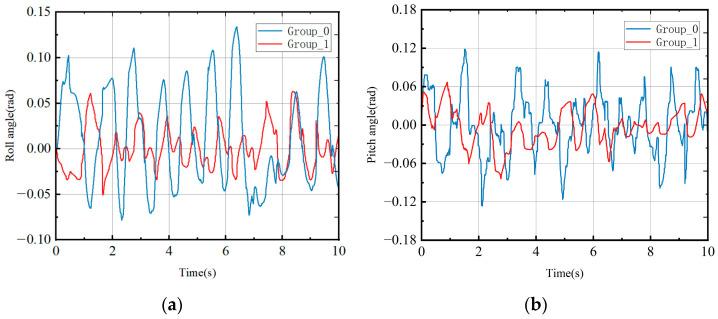
Changes in roll and pitch angles. (**a**) Roll angle. (**b**) Pitch angle.

The changes in the roll and pitch angles of the robot throughout the entire motion process were recorded, as depicted in Figure 6. It is apparent that Group_0 demonstrated relatively large amplitudes in both the roll and pitch angles throughout the entire motion cycle. This can be attributed to the substantial impact on the ground when the robot landed. In contrast, Group_1, which planned the swing leg trajectory in advance to minimize the ground impact during the transition from the swing phase to the support phase, exhibited a significant reduction in the changes in the roll and pitch angles. This enhancement in motion stability is a notable achievement resulting from the implementation of the hierarchical framework. This article employed the angle change variance as a criterion to evaluate the stability of the robot motion process. The variances in the roll and pitch angles for Group_0 were calculated as $2.5\times 10^{-3}$ and $3.1\times 10^{-3}$, respectively, while the variances for Group_1 were $8.9\times 10^{-4}$ and $2.1\times 10^{-3}$, respectively. Based on these results, it can be inferred that the layered framework proposed in this article enhances the motion stability of robots.

**Figure 7:**
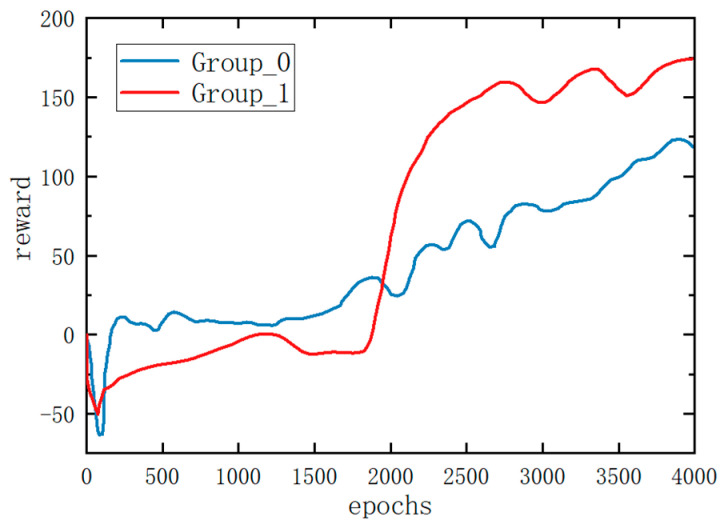
The reward curves of the two methods on flat ground. All policies were trained from scratch.

Figure 7 illustrates the average reward obtained by the two experimental groups. Group_1, trained using the hierarchical framework proposed in this paper, demonstrated a rapid increase in the reward value after approximately 1700 episodes. It surpassed Group_0 at around 2000 episodes and consistently maintained a higher reward value throughout the remaining training cycles, ultimately reaching a final reward value of approximately 175. In contrast, Group_0 achieved a final reward value of approximately 120. It is noteworthy that Group_1 obtained a reward value that is 45% higher than that of Group_0. These results provide compelling evidence that the hierarchical framework not only accelerates the training process but also leads to a substantial improvement in reward acquisition.

## 5. Conclusions

This paper presents a gait control framework utilizing hierarchical reinforcement learning for quadruped robots. The framework aims to enhance the training efficiency of the agent while maintaining the stability of traditional control methods.

The proposed framework comprises a high-level planner responsible for generating reference motion parameters and a low-level controller that calculates the required torques for each motor to achieve a trot gait. The algorithm is specifically designed with a predetermined state space, action space, and reward function. The current state is utilized as the input to obtain the motion parameters.

The low-level controller utilizes MPC to calculate the optimal foot-end forces for the support legs and subsequently determines the necessary output torques for each joint motor. For the swing legs, a combination of composite trajectory planning and a PD controller is employed to track the foot-end trajectory and compute the required output torques for each joint motor.

Simulation experiments were conducted to evaluate the effectiveness of the gait control framework using both deep reinforcement learning and hierarchical reinforcement learning. Experimental data were collected and comparatively analyzed to draw meaningful conclusions.

The main contents and conclusions of this paper are summarized as follows:

(1) In this study, kinematic and dynamic analyses of a quadruped robot were conducted. The D–H method was utilized to analyze the kinematics of a single leg in the quadruped robot, enabling the determination of the mapping relationship between the center of mass and the foot-end position, as well as the calculation of the Jacobian matrix. Following this, the dynamic models of the robot were simplified, resulting in the representation of a single-rigid-body state.
(2) An HRL framework was designed, consisting of a high-level planner and a low-level controller. Drawing inspiration from the concept of HRL, the complex control task of the quadruped robot was decomposed into two levels: high-level planning and low-level control. The high-level planner employed the DDPG algorithm to generate motion parameters for the robot. The leg motions were categorized into the support and swing phases. For the support leg, an optimization problem based on the MPC method was formulated to determine the optimal foot-end force. In the swing leg, a composite swing trajectory was employed to achieve the desired foot-end position at each time step, and a PD controller was utilized to generate a virtual foot-end force. The required torque for each joint motor was then calculated using the Jacobian matrix.
(3) The quadruped robot was simulated in Simulink, incorporating the aforementioned approaches. Simulation experiments were conducted using both deep reinforcement learning and hierarchical reinforcement learning methods. The results of these experiments confirmed the superiority of the hierarchical reinforcement learning method.

Our forthcoming experiments will be dedicated to fabricating a physical quadruped robot prototype with the ability to function within authentic environmental conditions. Our emphasis will encompass the amalgamation of diverse sensors to procure real-time environmental data. Furthermore, we intend to migrate the control algorithms established within simulated environments to the robot platform. This migration process will encompass the translation of algorithms into tangible hardware control interfaces.

## Figures and Tables

**Table 1:** Quadruped robot single-leg D–H parameter table.

Coordinate System	$a_{i-1}$	$\alpha_{i-1}$	$d_{i}$	$\theta_{i}$
0	$Trans(dx,dy,dz)R_{z}(-\frac{\pi }{2})$			
1	0	$\frac{\pi }{2}$	0	$\theta_{1}+\frac{\pi }{2}$
2	0	$\frac{\pi }{2}$	$L_{1}$	$\theta_{2}+\pi$
3	$L_{2}$	0	0	$\theta_{3}$
4	$L_{3}$	0	0	0

**Table 2:** Table of walking parameters for the quadruped robot.

Parameter	Definition	Value
$L_{x}$	Distance between front and rear rolling hip joints	141 mm
$L_{y}$	Distance between left and right rolling hip joints	78 mm
$L_{1}$	Hip joint connecting rod length	49 mm
$L_{2}$	Thigh length	125.6 mm
$L_{3}$	Calf length	136 mm
$\theta_{1}$	Roll hip angle	/
$\theta_{2}$	Pitch hip angle	/
$\theta_{3}$	Pitch knee angle	/

**Table 3:** Actuator specifications.

Entity	Value
Base mass	12 kg
Leg mass	0.4 × 4 kg
Number of joints	3 × 4
Max motor torque	30 N·m
Initial position	[0, 0, 0.175]
Initial motor angles	[0, 0.85, −1.7]

## Data Availability

Not applicable.
